# Systemic chemotherapy in addition to CRS‐HIPEC for colorectal peritoneal metastases: A critical systematic review on the impact on overall survival

**DOI:** 10.1002/jso.27849

**Published:** 2024-09-11

**Authors:** Teun B. M. van den Heuvel, Robin J. Lurvink, Koen P. B. Rovers, Irene E. G. van Hellemond, Ignace H. J. T. de Hingh

**Affiliations:** ^1^ Department of Surgery Catharina Hospital Eindhoven Eindhoven The Netherlands; ^2^ Department of Oncology and Developmental Biology (GROW), Faculty of Health, Medicine and Life Sciences Maastricht University Maastricht The Netherlands; ^3^ Department of Medical Oncology Catharina Hospital Eindhoven Eindhoven The Netherlands; ^4^ Department of Research & Development Netherlands Comprehensive Cancer Organisation (IKNL) Utrecht The Netherlands

**Keywords:** colorectal cancer, CRS‐HIPEC, overall survival, peritoneal metastases, systematic review, systemic chemotherapy

## Abstract

In patients with resectable colorectal peritoneal metastases, it is unclear whether systemic chemotherapy, in addition to cytoreductive surgery‐hyperthermic intraperitoneal chemotherapy (CRS‐HIPEC), improves overall survival (OS). This systematic review of 12 retrospective studies involving 3721 patients aimed to summarize the available evidence. Contradictory results were found regarding the effectiveness of neoadjuvant, adjuvant, and perioperative systemic therapies on OS, with a high risk of bias. Available evidence remains inconclusive, stressing the need for prospective, randomized trials, like the ongoing Dutch CAIRO6‐trial.

## 1 INTRODUCTION

Colorectal cancer is the third most prevalent cancer worldwide and is known to frequently disseminate to the peritoneum, resulting in peritoneal metastases (CPM).^1^, ^2^, ^3^ Depending on the extensiveness of peritoneal disease, patients may be eligible for treatment with cytoreductive surgery (CRS) with hyperthermic intraperitoneal chemotherapy (HIPEC), a curative intent treatment.^4^

Although the introduction of CRS with HIPEC has significantly improved the outcome of patients with limited CPM as compared to palliative systemic chemotherapy alone,^5^ recurrent diseases are common, negatively affecting overall survival (OS). It is currently unclear whether systemic chemotherapy, in addition to CRS with HIPEC, should play a role in the treatment of patients with resectable CPM and, if so, whether this should be given in a neoadjuvant setting, an adjuvant setting, or both. A potential benefit of neoadjuvant systemic chemotherapy is that it targets both systemic micrometastases and potentially decreases the peritoneal tumor load before CRS and HIPEC. Adjuvant systemic chemotherapy aims to target systemic micrometastases. Intuitively, both effects may result in better survival. On the other hand, the addition of systemic chemotherapy has some potential drawbacks. First, systemic chemotherapy may cause side effects, influencing the quality of life of patients. Second, neoadjuvant systemic chemotherapy delays the time to the CRS‐HIPEC procedure, potentially leading to disease progression and, as a result, irresectability. Also, response to neoadjuvant systemic chemotherapy may result in the development of fibrosis, which may complicate the intraoperative distinguishment between tumor tissue and scar tissue. Finally, more complications might occur after CRS‐HIPEC, due to a worse overall condition of patients.

Consequently, treatment strategies regarding the addition and the timing of systemic chemotherapy vary worldwide. These variations are a result of lacking prospective evidence on this topic, which may lead to patients being inadequately treated and to potentially unnecessary healthcare costs.^6^ Currently, the CAIRO6 trial is ongoing.^7^ This is a prospective randomized trial in which patients with resectable CPM receive either upfront CRS‐HIPEC or CRS‐HIPEC with perioperative systemic chemotherapy. The results of this trial are still to be expected. This systematic review was conducted to summarize the currently available evidence on the effect of systemic chemotherapy on OS in patients with resectable CPM who underwent CRS‐HIPEC.

## 2 MATERIALS AND METHODS

This systematic review was conducted according to the Preferred Reporting Items for Systematic Review and Meta‐Analyses (PRISMA) statement (Figure S1).^8^ Two researchers (T. H. and R. L.) performed the study selection (both abstract and full text screening), risk of bias assessment, data collection, and data synthesis independently. In case of disagreement between the two researchers, the study was discussed with a third researcher (K. R.) until a consensus was reached and a final decision was made.

### 2.1 Eligibility criteria

Original studies were deemed potentially eligible if they included patients with CPM who underwent CRS‐HIPEC and if the impact of neoadjuvant, adjuvant, or perioperative chemotherapy on OS was reported. Additionally, studies were considered ineligible if no comparison was made with patients who did not receive neoadjuvant, adjuvant, or perioperative chemotherapy, respectively. Neoadjuvant chemotherapy was defined as any systemic chemotherapy in patients with CPM before CRS‐HIPEC. Adjuvant chemotherapy was defined as any systemic chemotherapy intended as adjuvant chemotherapy by the authors in patients with CPM after CRS‐HIPEC. Perioperative systemic chemotherapy was defined as both neoadjuvant and adjuvant systemic chemotherapy in patients with CPM who underwent CRS‐HIPEC.

### 2.2 Search

The systematic search was performed on June 27, 2024, and encompassed PubMed/MEDLINE, EMBASE, and Cochrane databases without date or language restrictions. The comprehensive list of search terms is provided in Table S1.

### 2.3 Study selection

Titles and abstracts were screened for potential eligibility based on the predefined eligibility criteria, using Rayyan software. In case of conflicting opinions on, or uncertainty of eligibility, studies were evaluated after full text screening, as well as the studies that were deemed eligible by both researchers after title and abstract screening. Reference list screening of all potentially eligible manuscripts was performed to identify additional manuscripts that were potentially eligible for inclusion.

### 2.4 Data collection

Data collection was performed using a standardized list in which studies were divided based on the type of systemic treatment they evaluated: neoadjuvant or adjuvant chemotherapy or perioperative chemotherapy/multiple groups. This list contained data on the following items: year of publication, number of patients, inclusion period, study setting, information on the extensiveness of peritoneal metastases and completeness of CRS, regimen, and technique of intraperitoneal (IP) chemotherapy, the systemic regimen cohorts and timing of systemic chemotherapy, the number of patients per cohort, and the assessed outcome parameters.

If studies did not report numbers for median OS, these were deducted from the provided Kaplan−Meier graphs.

### 2.5 Methodology evaluation

Included studies were individually examined on their methodological quality using the Risk of Bias in Non‐Randomized Studies–of Interventions (ROBINS‐I) criteria.^9^

#### 2.5.1 Summary measures and synthesis of results

All expressions of OS, being median OS (months) or x‐year OS (%) were summarized. If studies reported on disease‐free survival (DFS), recurrence‐free survival, or progression‐free survival were available, these data were collected and assembled as DFS. All available survival data were narratively summarized for each chemotherapy subgroup. Since heterogeneity was present among included patients, interventions across studies, and outcome assessment, no meta‐analysis was performed.

## 3 RESULTS

### 3.1 Study selection

**Figure 1:**
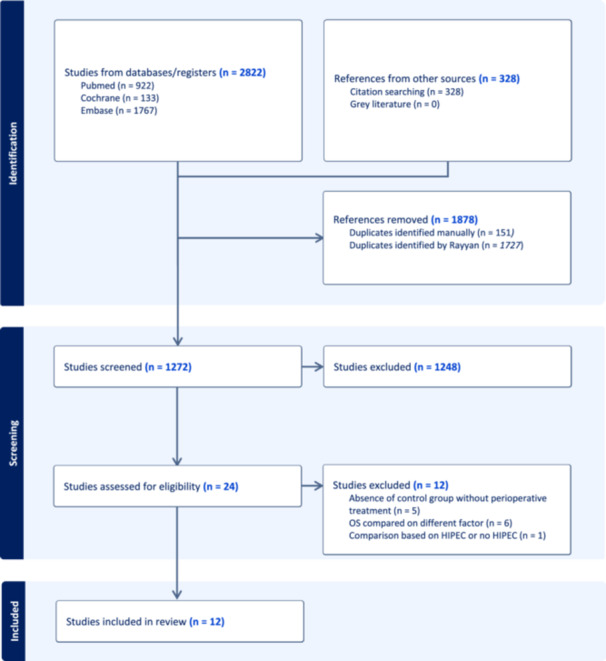
Flowchart of study selection. HIPEC, hyperthermic intraperitoneal chemotherapy; OS, overall survival.

The systematic search resulted in 2822 potential studies. After the abstract screening (Figure 1), 24 studies were considered potentially eligible. After full text screening, 12 studies were included: Beal (2020),^10^ Cashin (2023),^11^ Ceelen (2014),^12^ van Eden (2017),^13^ Kuijpers (2014),^14^ Maillet (2016),^15^ Passot (2012),^16^ Peng (2023),^17^ Repullo (2021),^18^ Rovers (2020),^19^ Tonello (2024),^20^ and Zhou (2021).^21^ Reasons for exclusion of potentially eligible studies are listed in Figure 1. Citations were searched for additional potentially eligible studies, but none were considered eligible.

### 3.2 Study characteristics

The included studies are listed in Table 1, presenting the study characteristics. The included studies were published between 2012 and 2024. All studies were retrospective in design, and five studies (45%) were multicenter. All studies included patients that were treated with CRS and HIPEC for histologically proven CPM, but between different studies heterogeneity was present regarding HIPEC‐strategy (open or closed, HIPEC‐regimen, duration), systemic treatment strategy (number of cycles, systemic chemotherapy regimen, timing, addition of targeted therapy), and outcome parameters.

**Table 1:** Overview of included studies.

Study (year)	Design			Cytoreductive surgery		HIPEC chemotherapy Regimen		Cohorts	*N*	Systemic therapy			Regimen	No of cycles				Targeted therapy	Outcome assessment	
	*N*	Inclusion period	Setting	Extent of PM	CC score		HIPEC technique			NACT	ACT	POCT		Minimum	Maximum		Mean		Outcome	Interval
Neoadjuvant chemotherapy (NACT)																				
Beal (2020)	298	2000−2017	Multicenter, retrospective	PCI 12.9 (±8.2)	0: 71% 1: 16% ≥2: 12%	MMC: 97% Oxaliplatin: 3%	N/A	No NACT^a^	102	0 (0%)	16 (33%)	‐	‐	‐				‐	OS DFS	HIPEC‐death HIPEC‐recurrence or death
								NACT^a^	196	196 (100%)	62 (40%)	‐	Mono 15 (8%) Doublet 149 (76%) Triplet 5 (3%) Other 27 (14%)	N/A		N/A	N/A	12 (6%) 89 (45%) 5 (3%) 3 (2%)		
Cashin (2022)	708	1991−2018	Multicenter, retrospective	PCI 10. (±7.1)	0: 92% 1: 7% ≥2: 2%	MMC: 43% Oxaliplatin ± irinotecan: 56% Other: 1%	N/A	No NACT^a^	354	0 (0%)	66 (19%)	‐	‐	‐				‐	OS DFS	N/A
								NACT^a^	354	354 (100%)	74 (21%)	‐	N/A	N/A		N/A	N/A	N/A		
Ceelen (2014)	166	2002−2012	Monocenter, retrospective	Region count 4 (2−5)	0: 47% 1: 40% 2: 13%	MMC: 26% Oxaliplatin: 74%	Open	No NACT^a^	105	0 (0%)	49 (47%)	‐	‐	‐				‐	OS	HIPEC‐death
								NACT^a^	61	61 (100%)	34 (56%)	‐	Doublet 61 (100%)	6	N/A		N/A	26 (43%)		
Passot (2012)	120	1991−2010	Monocenter, retrospective	PCI 8.2 (±3.5)	0: 77% 1: 9% ≥2: 14%	MMC MMC + oxaliplatin MMC + irinotecan	Closed	No NACT^a^	30	0 (0%)	77 (63%)^a^	‐	‐	‐				‐	OS	HIPEC‐death
								NACT^a^	90	90 (100%)		‐	Mono 22 (24%) Doublet 63 (70%) Other 5 (6%)	N/A		N/A	7.4	0 (0%) 17 (19%) 0 (0%)		
Peng (2024)	251	2012−2019	Monocenter, retrospective	PCI ≤10: 69% >10: 31%	0−1: 38% ≥2: 62%	None: 58% 5FU Oxaliplatin Loplatin	Closed	No NACT^a^	209	0 (0%)	N/A	‐	‐	‐				‐	OS	N/A
								NACT^a^	42	42 (100%)	N/A	‐	Doublet 29 (69%) Triplet 13 (31%)	N/A		N/A	N/A	In total 12 (29%)		
Zhou (2021)	52	2017−2019	Monocenter, retrospective	PCI 11.9 (±5.6)	0‐1: 60% ≥2: 40%	Oxaliplatin + faltitrexed: 56% Oxaliplatin + lobaplatin + faltitrexed: 44%	Closed	No NACT^a^	32	0 (0%)	28 (88%)	‐	‐	‐				‐	OS	HIPEC‐death
								NACT^a^	20	20 (100%)	16 (80%)	‐	Mono 1 (5%) Doublet 19 (95%)	3		N/A	N/A	0 (0%) 6 (30%)		
Adjuvant chemotherapy (ACT)																				
Cashin (2022)	778	1991−2018	Multicenter, retrospective	PCI 9.6 (±6.9)	0: 95% 1: 4% ≥2: 1%	MMC: 37% Oxaliplatin ± irinotecan: 62% Other: 1%	N/A	No ACT^a^	389	285 (73%)	0 (0%)	‐	‐	‐				‐	OS DFS	N/A
								ACT^a^	389	285 (73%)	389 (100%)	‐	N/A	N/A		N/A	N/A	N/A		
Maillet (2016)	231	2004−2012	Multicenter, retrospective	PCI 1−6: 36% 7−12: 32% 13−19: 20% >19: 12%	0: 100%	MMC: 16% Oxaliplatin: 48% Oxaliplatin + irinotecan: 31% Other: 5%	Open 54% Closed 46%	No ACT^a^	70	N/A	0 (0%)	‐	‐	‐				‐	OS DFS	HIPEC‐death HIPEC‐recurrence or death
								ACT^a^	151	116 (77%)	151 (100%)	‐	Mono 8 (5%) Doublet 142 (94%) Triplet 1 (1%)	1		N/A	N/A	4 (3%) 75 (50%) 0 (0%)		
Rovers (2020)	284	2005−2017	Multicenter, retrospective	N/A	N/A	MMC Oxaliplatin	Open	No ACT^a^	142	0 (0%)	0 (0%)	‐	‐	‐				‐	OS	HIPEC‐death
								ACT^a^	142	0 (0%)	142 (100%)	‐	Mono 9 (6%) Doublet 75 (53%) Unknown 58 (41%)	N/A		N/A	N/A	0 (0%) 0 (0%) 0 (0%)		
Perioperative chemotherapy or multiple groups (POCT)																				
Kuijpers (2014)	73	2004−2012	Monocenter, retrospective	Region count 3.0 (2.6−3.5)	0−1: 87% 2: 13%	MMC: 100%	Open	No POCT^a^	16	0 (0%)	0 (0%)	0 (0%)	‐	‐				‐	OS DFS	HIPEC‐death HIPEC‐recurrence or death
								POCT^a^	55	14 (26%)	32 (58%)	9 (16%)	N/A	N/A		N/A	N/A	N/A		
Repullo (2021)	123	2008−2017	Multicenter, retrospective	PCI 6 (1−25)	0−1: 100%	Oxaliplatin: 98% MMC: 2%	Open 58% Closed 42%	No POCT^a^	69	‐	‐	0 (0%)	‐	‐				‐	OS DFS	HIPEC‐death HIPEC‐recurrence or death
								POCT^a^	56	‐	‐	56 (100%)	N/A	5		N/A	N/A	N/A		
Tonello (2024)	367	2013−2018	Multicenter, retrospective	PCI 9.5 (±6.3)	0: 84% 1: 16%	MMC Cisplatin Oxaliplatin	N/A	No POCT^a^	73	0 (0%)	0 (0%)	0 (0%)	‐	‐				‐	OS DFS	HIPEC‐death HIPEC‐recurrence or death
								POCT^a^	294	119 (41%)	106 (36%)	69 (23%)	Mono 12 (4%) Doublet 239 (82%) Triplet 33 (11%) Unknown 10 (3%)	N/A		N/A	N/A	In total 172 (59%)		
van Eden (2017)	280	2004−2015	Monocenter, retrospective	Region count 0: 4% 1−2: 47% 3−5: 41% 6−7: 8%	0−1: 91% ≥2: 9%	MMC: 86% Oxaliplatin 14%	N/A	No POCT^a^	33	0 (0%)	0 (0%)	‐	‐	‐				‐	OS DFS	Diagnosis‐death Diagnosis‐recurrence
								NACT or POCT^a^	78	78% (100%)	26 (33%)	‐	Doublet 100%	<6 CAPOX/ <8 FOLFOX: 23 (29%) >6 CAPOX/ >8 FOLFOX: 55 (71%)				0 (0%) 0 (0%)		
								ACT^a^	169	0 (0%)	115 (68%)	‐	Doublet 92% Unknown 8%	0 cycles: 54 (32%) <6 CAPOX/ <8 FOLFOX: 24 (14%) >6 CAPOX/ >8 FOLFOX: 83 (49%) Unknown: 8 (5%)				0 (0%) 0 (0%)		

Abbreviations: ACT, adjuvant systemic chemotherapy; CC score, completeness of cytoreduction score; HIPEC, hyperthermic intraperitoneal chemotherapy; MMC, mitomycin C; N/A, not available; NACT, neoadjuvant systemic chemotherapy; PCI, peritoneal carcinomatosis index; PM, peritoneal metastases; POCT, perioperative systemic chemotherapy.

a Reported for both groups together.

A total of 3721 patients were included in all studies, varying from 52 to 1486 patients per study. Of these, 905 patients received neoadjuvant systemic chemotherapy and were compared to 984 patients who did not receive neoadjuvant systemic chemotherapy; 957 patients received adjuvant systemic chemotherapy and were compared to 707 patients who did not receive adjuvant systemic chemotherapy; 235 patients received perioperative systemic chemotherapy and were compared to 191 patients who did not receive perioperative systemic chemotherapy. The performed systemic treatment strategies per study were presented in Table 1. Three studies did not provide information on systemic chemotherapy regimens and on the usage of targeted therapy. In all of the studies describing systemic chemotherapy strategy, doublet systemic chemotherapy was mostly used (varying from 53% to 100% of patients with systemic chemotherapy). Targeted therapy was described in nine studies, of which two studies in which no targeted therapy was used. In the remaining seven studies, usage of targeted therapy varied between 19% and 59%.

### 3.3 OS

#### 3.3.1 Neoadjuvant systemic chemotherapy

The OS outcomes from studies on neoadjuvant systemic chemotherapy are presented in Table 2A. In total, 984 patients received upfront CRS‐HIPEC, and 905 patients received neoadjuvant systemic chemotherapy before CRS‐HIPEC. Four studies described a statistically significant improved OS in patients treated with neoadjuvant systemic chemotherapy: Beal (2020), Ceelen (2014), Passot (2012), and Zhou (2021).^10^, ^12^, ^16^, ^21^ Two studies did not find a significant difference: Cashin (2023) and Peng (2023).^11^, ^17^

**Table 2:** Overview of results on overall survival per study.

A. Neoadjuvant systemic chemotherapy											
**Study**		**Treatment groups**			**Overall survival**					**Univariable**	**Multivariable**
	Median follow‐up		*n*	%	Median	1‐year	2‐year	3‐year	5‐year	*p* Value	HR (95% CI)
Beal (2020)	19	Yes No	196 102	66 34	33 (CI not reported) 22 (CI not reported)					0.044	0.69 (0.48–0.99)
Cashin (2022)	N/A	Yes No	354 354	50 50	35 (31−39) 37 (33–43)					0.46	1.08 (0.88–1.32)
Ceelen (2014)	18	NACT NACT + bevacizumab No	35 26 105	21 16 63	22 (13–31) 39 (18–60) 25 (19–31)					0.021 0.019	0.31 (0.12−0.83)
Passot (2012)	59 (SD ± 48)	Yes No	90 30	75 25	36^a^ 23^a^					0.040	
Peng (2023)	35 (IQR 29–40)	Yes No	42 209	17 83	28^a^ 19^a^					0.88	1.03 (0.71−1.49)
Zhou (2021)	19	Yes No	20 32	39 62	N/A 20^a^		67% 32%			0.033	0.55 (0.22−1.39)

**B. Adjuvant systemic chemotherapy**											
**Study**		**Treatment groups**			**Overall survival**					**Univariable**	**Multivariable**
	Median follow‐up		*n*	%	Median	1‐year	2‐year	3‐year	5‐year	Log‐rank *p*	HR (95% CI)
Cashin (2022)	N.A.	Yes No	389 389	50 50	46 (39–56) 37 (33–42)					0.022	0.79 (0.64−0.97)
Maillet (2016)	34	Yes No	151 70	68 32	49 (CI not reported) 43 (CI not reported)					0.93	1.13 (0.70−1.84)
Rovers (2020)	26 (IQR 16–47)	Yes No	142 142	50 50	39 (21−111) 25 (15−58)	92% 81%		55% 41%	35% 22%	0.003	0.66 (0.49−0.88)

**C. Combination or perioperative systemic chemotherapy**											
**Study**		**Treatment groups**			**Overall survival**					**Univariable**	**Multivariable**
	Median follow‐up		*n*	%	Median	1‐year	2‐year	3‐year	5‐year	Log‐rank *p*	HR (95% CI)
Kuijpers (2014)	47 (95% CI 43–51)	Yes No	55 16	77 23	30 (19−41) 14 (3−25)			45% 16%		0.015	
Repullo (2021)	54 (SD ± 5)	Yes No	56 69	45 55	43 (31−58) 72 (53−88)	98% 97%		59% 77%	35% 56%	0.155	1.46 (0.87−2.47)
Tonello (2024)	39 (95% CI 35–49)	Yes No NACT Adjuvant Perioperative	294 73 119 106 69	80 20 32 29 19	39 (33−48) 55 (22−N/A) 39 (30−48) 38 (28−54) 43 (30−57)					0.56 Ref. 0.71 0.88	Ref. 0.85 (0.52−1.40) Ref. 0.76 (0.49−1.19) 0.79 (0.51−1.24)
Van Eden (2017)	30 (IQR 17–53)	NACT/perioperative Adjuvant No	23/55 169 33	28 60 12	37 (21–80) 43 (26–96) 34 (20–54)					0.19	2.52 (1.48−4.29)

Abbreviations: CI, confidence intervals; HR, hazard ratio; IQR, interquartile range; N/A, not available; NACT, neoadjuvant chemotherapy; SD, standard deviation.

a Median overall survival was not available in numbers, but was measured using the Kaplan–Meier curve, influencing accuracy.

Beal (2020), Cashin (2023), and Ceelen (2014) provided median OS.^10^, ^11^, ^12^ Beal found an improved OS in patients treated with neoadjuvant systemic chemotherapy compared to upfront CRS‐HIPEC, while the other two did not (Beal et al.: 33 vs. 22 months, *p* = 0.044/Cashin et al.: 35 vs. 37 months, *p* = 0.46/Ceelen: 22 vs. 25 months, *p* = 0.021, respectively). Additionally, the study of Ceelen et al. found a survival benefit when a combination of neoadjuvant systemic chemotherapy and bevacizumab was administered compared to upfront CRS‐HIPEC (39 vs. 25 months, *p* = 0.019, respectively).

Passot (2012) and Peng (2023) only provided Kaplan−Meier graphs, of which median OS could be deducted. The study of Passot et al. reported a survival benefit in favor of treatment with neoadjuvant systemic chemotherapy compared to upfront surgery (median OS 37 vs. 23 months, *p*‐value 0.040, respectively).^16^ The study of Peng et al. did not find a survival benefit (median OS 28 vs 19 months, *p*‐value 0.88, respectively).^17^

The trial of Zhou (2021) provided 2‐year OS rates and reported a survival benefit in favor of treatment with neoadjuvant systemic chemotherapy compared to no neoadjuvant systemic chemotherapy (67% vs. 32%, *p* = 0.044, respectively).^21^

#### 3.3.2 Adjuvant systemic chemotherapy

The OS outcomes from studies on adjuvant systemic chemotherapy are presented in Table 2B. In total, 707 patients did not receive adjuvant systemic chemotherapy after CRS‐HIPEC, and 957 patients did receive adjuvant systemic chemotherapy after CRS‐HIPEC. Two studies described an OS benefit in patients treated with adjuvant systemic chemotherapy: Cashin (2022) and Rovers (2020).^11^, ^19^ Two studies did not find a significant difference: Maillet (2016) and van Eden (2017).^13^, ^15^

The studies of Cashin (2023) and Rovers (2020) reported a median OS benefit in patients with adjuvant systemic chemotherapy compared to no adjuvant systemic chemotherapy (Cashin 46 vs. 37 months, *p* = 0.022/Rovers 39 vs. 25 months, *p* = 0.003, respectively).^11^, ^19^

The trials of Maillet (2016) and van Eden (2017) did not find a significant OS benefit for adjuvant systemic chemotherapy compared to no adjuvant systemic chemotherapy (Maillet 49 vs. 43 months, *p* = 0.93/van Eden 43 vs. 34 months, *p* = 0.19, respectively).^13^, ^15^

#### 3.3.3 Combination or perioperative systemic chemotherapy

The OS outcomes from studies on perioperative systemic chemotherapy are presented in Table 2C. Perioperative systemic chemotherapy was investigated in four studies: Kuijpers (2014), Repullo (2021), Tonello (2024), and van Eden (2017).^13^, ^14^, ^18^, ^20^ Patients receiving CRS‐HIPEC without systemic chemotherapy (*n* = 191) were compared to patients receiving perioperative systemic chemotherapy (*n* = 133). An association between perioperative systemic chemotherapy and improved OS was found in one study.^14^ However, studies on perioperative systemic chemotherapy often combined perioperative systemic chemotherapy and neoadjuvant or adjuvant systemic chemotherapy in one group.

The study of Kuijpers (2014) presented a survival benefit for perioperative systemic chemotherapy compared to no chemotherapy, based on median and 3‐year OS (30 vs. 14 months, 45% vs. 16%, *p* = 0.015, respectively). Within the systemic chemotherapy group, no distinction was made between neoadjuvant, adjuvant, or perioperative systemic chemotherapy.^14^

The studies of Repullo (2021) and Tonello (2024) compared patients who had received any systemic chemotherapy (neoadjuvant, adjuvant, or perioperative) to patients who had not received systemic chemotherapy. OS was presented as median OS and in both studies no survival benefit was found for systemic chemotherapy compared to no systemic chemotherapy (Repullo 43 vs. 72 months, *p* = 0.115/Tonello 39 vs. 55 months, *p* = 0.56, respectively).^18^, ^20^

Van Eden (2017) combined patients with neoadjuvant and perioperative systemic chemotherapy in one group and compared these to patients who had not received systemic chemotherapy. No significant benefit in median OS was found between these two groups (37 vs. 34 months, *p* = 0.19, respectively).^13^

### 3.4 DFS

The DFS outcomes are presented in Table 3. Two studies on neoadjuvant systemic chemotherapy reported DFS, but they did not find a significant difference in DFS.^10^, ^11^

**Table 3:** Overview of results of studies that reported on disease free survival.

Study		*N*	%	Disease free survival	Log‐rank *p*	HR (95% CI)
A. Neoadjuvant systemic therapy						
Beal (2020)	Yes	196	66	14 (CI not reported)	0.456	1.04 (0.74−1.47)
	No	102	34	13 (CI not reported)		
Cashin (2022)	Yes	354	50	12 (11−14)	0.66	
	No	354	50	13 (11−14)		1.04 (0.87−1.25)
B. Adjuvant systemic therapy						
Cashin (2022)	Yes	389	50	13 (12−15)	0.030	
	No	389	50	11 (10−13)		0.83 (0.70−0.98)
Maillet (2016)	Yes	151	68	13 (CI not reported)	0.77	0.97 (0.64−1.48)
	No	70	32	10 (CI not reported)		
C. Perioperative or combined systemic therapy						
Kuijpers (2014)	Yes	55	77	15 (14−16)		
	No	16	23	4 (1−7)	0.024	
Repullo (2021)	Yes	56	45	11 (9−16)	0.035	1.22 (0.78−1.92)
	No	69	55	17 (9−25)		
Tonello (2024)	Yes	294	80	13 (11−14)		
	No	73	20	16 (11−23)	0.50	0.79 (0.56−1.11)
	NACT	119	32	10 (8−12)	Ref.	Ref.
	Adjuvant	106	29	15 (13−18)	0.0030	0.57 (0.40−0.79)
	Perioperative	69	19	13 (10−15)	0.1680	0.80 (0.56−1.13)
Van Eden (2017)	NACT/perioperative	23/55	28	20^a^	0.29	Ref.
	Adjuvant	169	60	16^a^		2.01 (1.26−3.91)
	No	33	12	22^a^		2.01 (1.21−3.33)

Abbreviations: CI, confidence intervals; HR, hazard ratio; NACT, neoadjuvant chemotherapy.

a Disease free survival was not available in numbers but was measured using the Kaplan−Meier curve, influencing accuracy.

Two studies on adjuvant systemic chemotherapy reported DFS (10, 14). One of these studies found an improved DFS for patients treated with CRS‐HIPEC followed by adjuvant systemic chemotherapy compared to CRS‐HIPEC without adjuvant systemic chemotherapy (13 vs. 11 months, *p* = 0.030) (10). The other study found no difference in DFS (14).

Two studies on perioperative systemic chemotherapy reported DFS (13, 17), of which one found an improved DFS in patients treated with perioperative systemic chemotherapy (15 vs. 4 months, *p* = 0.024, respectively) (13). The second study found no difference in DFS (17).

Finally, two studies on a combination of neoadjuvant, adjuvant, and/or perioperative systemic chemotherapy reported DFS, but neither found a DFS difference (12, 19).

### 3.5 Quality criteria

**Figure 2:**
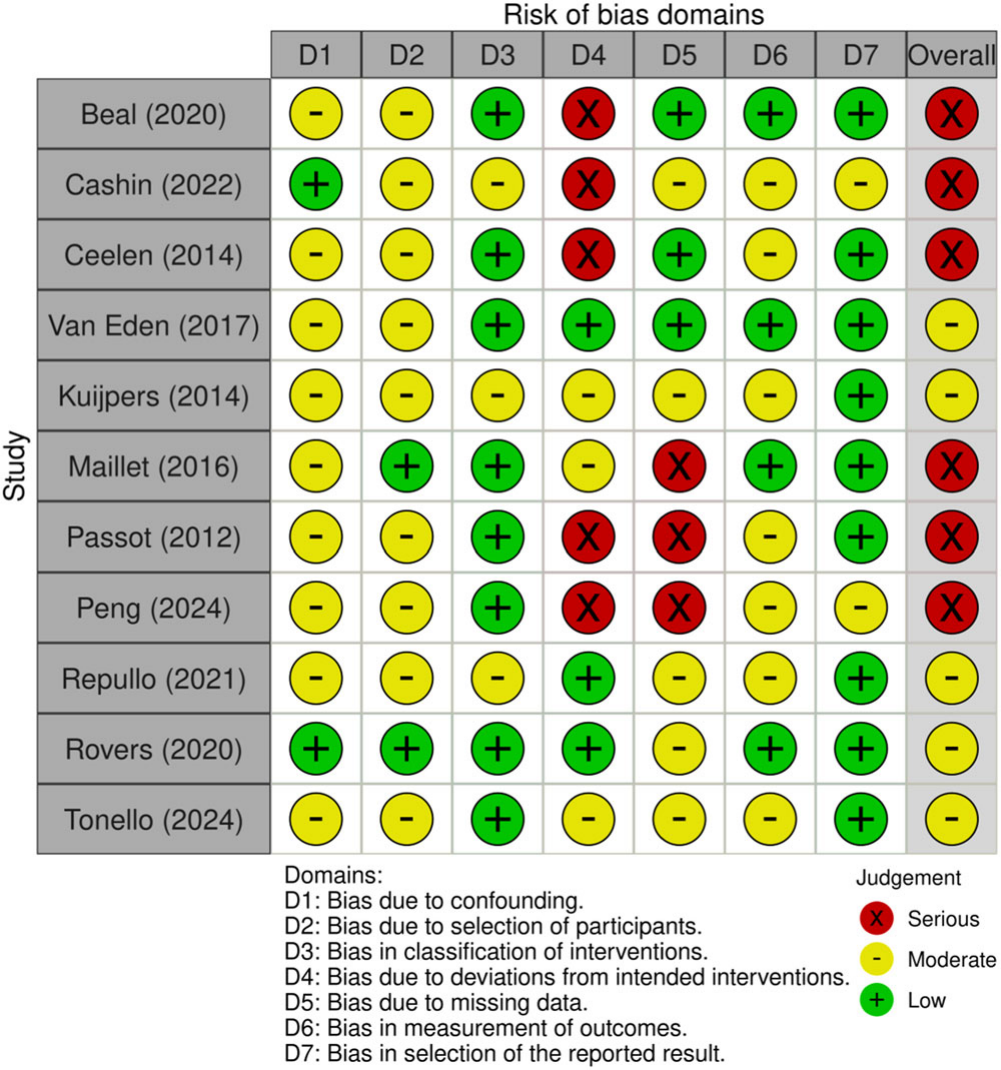
ROBIN's criteria per study.

The ROBINS‐I criteria were used to assess the risk of bias in included studies (Figure 2).^9^ All studies were considered to have at least a moderate risk of bias due to their retrospective design, introducing selection bias, although two studies performed propensity score analysis to correct for this. Four studies described or failed to describe relevant co‐interventions (e.g., administering systemic chemotherapy at a different time point than investigated) of the included patients. Three studies failed to describe the type and number of systemic regimens used.

## 4 DISCUSSION

This systematic review aimed to evaluate the currently available evidence on the impact of systemic chemotherapy in addition to CRS‐HIPEC on OS in patients with CPM. No completed randomized controlled trials were found. Therefore, only retrospective cohort studies were included in this review, resulting in clinical heterogeneity among the patients in the included studies and low methodological quality.

Among 12 included studies, six studies found a statistically significant improved OS in patients treated with CRS‐HIPEC and systemic chemotherapy: four^10^, ^12^, ^16^, ^21^ out of seven studies on neoadjuvant systemic chemotherapy^11^, ^17^, ^20^; three^11^, ^19^ out of five studies on adjuvant systemic chemotherapy^13^, ^15^, ^20^; and one^14^ out of four studies on perioperative systemic chemotherapy.^13^, ^18^, ^20^

Regarding DFS, two^11^, ^14^ out of seven studies^10^, ^13^, ^15^, ^18^, ^20^ found an improved DFS in patients who were treated with CRS‐HIPEC and systemic chemotherapy as compared to patients who had not received systemic chemotherapy in addition to CRS‐HIPEC. Only Cashin et al. and Kuijpers et al. concluded a significant benefit of respectively adjuvant and perioperative systemic chemotherapy on DFS. This disparity in conclusions illustrates the current problem regarding the question whether systemic chemotherapy in addition to CRS‐HIPEC is beneficial or not.^11^, ^14^

### 4.1 Clinical heterogeneity

Clinical heterogeneity among the included studies was mainly caused by variation in systemic chemotherapy regimens, the (threshold used for) extensiveness of CPM, and the use of co‐interventions. Variations in systemic chemotherapy regimens existed between, but also within, studies, which consisted of differences in the number of cycles administered, the use of mono‐, doublet, and triplet systemic chemotherapy, and the addition of targeted therapy. Also, several studies failed to report this information.^11^, ^14^, ^18^ This variation in treatment with systemic chemotherapy is very common worldwide due to the lack of studies to guide treatment choices.^4^, ^20^ Previous research found doublet or triplet therapy to be more effective than monotherapy, and the introduction of targeted therapy has also increased prognosis.^22^, ^23^ Thus, it is disputable whether the absence of an association between improved OS and systemic chemotherapy is substantiated with this degree of clinical heterogeneity. This emphasizes the need for consensus on the optimal systemic treatment for these patients, once evidence has been established to support treatment with systemic chemotherapy in CPM patients who are candidates for CRS‐HIPEC.

This optimal treatment might also be dependent of the molecular characteristics of tumors. In recent years, molecular diagnostics have become more frequently used, and thus more important, in the decision‐making of treatment in patients with CPM. The acquired information on molecular characteristics, as well as on mutational status and microsatellite status, may be used to divide tumors into subgroups and to adjust treatment strategies based on these tumor characteristics.^24^, ^25^

Another source of clinical heterogeneity occurred due to the different methods used to assess the extensiveness of PM: most studies used the peritoneal carcinomatosis index,^10^, ^11^, ^15^, ^17^, ^18^, ^20^, ^21^ but several studies used other scoring systems, such as the Gilly score,^16^ Dutch seven region count,^13^, ^14^ or the nine region count,^12^ and one study did not report the extensiveness of PM.^19^

Additionally, different thresholds for resectable disease and curative intent treatment were used between studies, with maximum PCI scores ranging from 15 to 25 to include patients for curative intent treatment.^18^, ^20^ Given that the extensiveness of PM is known to strongly affect prognosis, these differences among studies seriously affect the interpretation and comparison of their results.^26^

Finally, the lack of information on the use of co‐interventions caused clinical heterogeneity, in particular on the use of additional systemic chemotherapy at a different time‐point than investigated. This was the case for all studies on neoadjuvant systemic chemotherapy that compared patients who had received neoadjuvant systemic chemotherapy followed by CRS‐HIPEC to patients who had received upfront CRS‐HIPEC.^10^, ^11^, ^12^, ^16^, ^17^, ^21^ Several patients in the latter group did receive adjuvant systemic chemotherapy, or this information was not available in the study. The same occurred in two out of three studies on adjuvant systemic chemotherapy that compared patients who had received CRS‐HIPEC followed by adjuvant systemic chemotherapy with patients who were not treated with adjuvant systemic chemotherapy after CRS‐HIPEC.^11^, ^15^ In these two studies, several patients of the latter group were treated with neoadjuvant systemic chemotherapy.

Thus, the absence of an association between systemic chemotherapy and improved survival might be attributed to the use of these co‐interventions.

### 4.2 Prospective data

Previously, the prospective single‐arm COMBATAC trial evaluated perioperative FOLFOX/FOLFIRI plus Cetuximab in 25 patients with CPM eligible for CRS‐HIPEC, but this trial was discontinued in 2014 due to insufficient patient accrual.^27^ They concluded that the perioperative treatment was both feasible and safe and reported a median OS of 23 months. To the best of our knowledge, there is only one prospective trial currently ongoing that investigates whether CRS‐HIPEC combined with systemic chemotherapy results in an improved OS as compared to CRS‐HIPEC alone: the Dutch CAIRO6‐trial.^7^ In this randomized controlled trial, patients with resectable CPM are being allocated to either CRS‐HIPEC without perioperative systemic chemotherapy or CRS‐HIPEC with perioperative systemic chemotherapy. This trial aims to include 358 CPM patients and should provide valuable evidence on the question of whether perioperative systemic chemotherapy leads to an improved OS. In 2023, Phase 2 results were published, showing that perioperative systemic therapy is both feasible and safe: CRS‐HIPEC was as equally often performed in patients with neoadjuvant systemic chemotherapy, as it was in patients without systemic chemotherapy. Furthermore, surgical complications were equally prevalent, and quality of life was comparable to patients with systemic chemotherapy. The expected potential drawbacks of perioperative systemic chemotherapy were thus disproved by this Phase 2 trial.^28^ As presented during the PSOGI‐conference in 2023, the accrual of the CAIRO6‐trial is getting to an end.^7^

### 4.3 Limitations

As previously stated, the main limitation of the currently available evidence is the lack of prospective or randomized data. All included studies were based on retrospective data, resulting in selection bias in patients selected for treatment with systemic chemotherapy. Also, it is unclear how many patients were not able to undergo CRS‐HIPEC due to disease progression during neoadjuvant systemic chemotherapy, impeding the addition of the intention‐to‐treat analyses.

Some of the included studies were also limited by their sample size,^15^, ^17^, ^21^ as their results showed a trend toward significance in favor of treatment with systemic chemotherapy combined with CRS‐HIPEC. However, potentially because of their sample size, no significant survival benefit could be distinguished.

Finally, outcome reporting differed among studies, impeding the comparison of results. Several studies did not present median OS but only illustrated OS through a Kaplan–Meier graph or as 1‐, 2‐, 3‐, or 5‐year OS, resulting in less exact numbers for median OS.

## 5 CONCLUSIONS

In patients with resectable CPM, the currently available evidence on the association between neoadjuvant, adjuvant, and perioperative systemic chemotherapy on OS solely consists of retrospective cohort studies with conflicting results and a high probability of selection bias. The role of neoadjuvant, adjuvant, and perioperative systemic chemotherapy in patients with resectable CPM, therefore, remains unclear. Prospective, randomized trials are needed to investigate whether perioperative systemic chemotherapy should be standard practice in patients with resectable CPM. The ongoing Dutch CAIRO6 randomized controlled trial should provide the much‐needed evidence on this question.

## SYNOPSIS

Worldwide, different strategies are used in the treatment of patients with resectable peritoneal metastases of colorectal origin. This systematic review evaluated the available evidence on the impact of systemic chemotherapy in addition to CRS‐HIPEC in these patients.

## Supporting information

Appendix – Supplementary figure 1 ‐ PRISMA checklist.

Appendix – Supplementary table 1 – Search.

## Data Availability

The data that support the findings of this study are available from the corresponding author (T. H.) upon reasonable request.
