# Supplementary material for: Systemic chemotherapy in addition to CRS‐HIPEC for colorectal peritoneal metastases: A critical systematic review on the impact on overall survival
Source: J Surg Oncol. 2024 Sep 11;130(6):1378–89. doi: 10.1002/jso.27849 (PMC11825997; doi:10.1002/jso.27849)
Supplement: Supplementary file 2 — Appendix – Supplementary table 1 – Search. [file JSO-130-1378-s002.docx]

| Supplementary Table 1A – Search strategy PubMed | | |
| --- | --- | --- |
|  | MeSH-term | Free search |
| #1 Colorectal carcinoma | Colorectal Neoplasms OR Colonic Neoplasms OR Rectal Neoplasms | Colorectal cancer OR Colorectal carcinoma OR Colon cancer OR Colon carcinoma OR Rectal cancer OR Rectal carcinoma OR Colorectal malignancies OR Colorectal neoplasia OR Colorectal neoplasm OR Rectal malignancies OR Rectal neoplasia OR Rectal neoplasm |
| #2 Peritoneal metastases |  | Peritoneal metastases OR Peritoneal metastasis OR Peritoneal carcinomatosis OR Peritoneum OR Carcinomatosis OR Peritoneal surface malignancies |
| #3 CRS-HIPEC | Cytoreduction Surgical Procedures OR Hyperthermic Intraperitoneal Chemotherapy | CRS OR Cytoreductive surgery OR Debulking OR Cytoreduction OR Hyperthermic intraperitoneal chemotherapy OR HIPEC OR Intraperitoneal chemotherapy |
| #4 Systemic therapy | Chemotherapy, Adjuvant OR Neoadjuvant Therapy | Perioperative chemotherapy OR Adjuvant chemotherapy OR Neoadjuvant chemotherapy OR Perioperative systemic therapy OR Adjuvant systemic therapy OR Neoadjuvant systemic therapy OR Systemic therapy OR Chemotherapy |
| #5 Outcomes | Survival OR Disease-Free Survival OR Survival Analysis | Survival OR Disease-free survival OR Survival analysis |
| Search: (#1 MeSH-term OR #1 Free search) AND (#2 MeSH-term OR #2 Free search) AND (#3 MeSH-term OR #3 Free search) AND (#4 MeSH-term OR #4 Free search) AND (#5 MeSH-term OR #5 Free search) | | |
| Supplementary Table 1B – Search strategy Cochrane | | |
|  | MeSH-term | Free search |
| #1 Colorectal carcinoma | Colorectal Neoplasms OR Colonic Neoplasms OR Rectal Neoplasms | Colorectal cancer OR Colorectal carcinoma OR Colon cancer OR Colon carcinoma OR Rectal cancer OR Rectal carcinoma OR Colorectal malignancies OR Colorectal neoplasia OR Colorectal neoplasm OR Rectal malignancies OR Rectal neoplasia OR Rectal neoplasm |
| #2 Peritoneal metastases |  | Peritoneal metastases OR Peritoneal metastasis OR Peritoneal carcinomatosis OR Peritoneum OR Carcinomatosis OR Peritoneal surface malignancies |
| #3 CRS-HIPEC | Cytoreduction Surgical Procedures OR Hyperthermic Intraperitoneal Chemotherapy | CRS OR Cytoreductive surgery OR Debulking OR Cytoreduction OR Hyperthermic intraperitoneal chemotherapy OR HIPEC OR Intraperitoneal chemotherapy |
| #4 Systemic therapy | Chemotherapy, Adjuvant | Perioperative chemotherapy OR Adjuvant chemotherapy OR Neoadjuvant chemotherapy OR Perioperative systemic therapy OR Adjuvant systemic therapy OR Neoadjuvant systemic therapy OR Systemic therapy OR Chemotherapy |
| #5 Outcomes | Survival OR Disease-Free Survival OR Survival Analysis | Survival OR Disease-free survival OR Survival analysis OR overall survival |
| Search: (#1 MeSH-term OR #1 Free search) AND (#2 MeSH-term OR #2 Free search) AND (#3 MeSH-term OR #3 Free search) AND (#4 MeSH-term OR #4 Free search) AND (#5 MeSH-term OR #5 Free search) | | |
| Supplementary Table 1C – Search strategy EMBASE | | |
|  | MeSH-term | Free search |
| #1 Colorectal carcinoma |  | Colorectal tumor OR Colon tumor OR Rectum tumor OR Colorectal cancer OR Colorectal carcinoma OR Colon cancer OR Colon carcinoma OR Rectum cancer OR Rectum carcinoma |
| #2 Peritoneal metastases |  | Peritoneum metastasis OR Carcinomatous peritonitis OR Peritoneum OR Carcinomatosis OR Peritoneum cancer |
| #3 CRS-HIPEC |  | Cytoreductive surgery OR Hyperthermic intraperitoneal chemotherapy OR Debulking OR Cytoreduction OR Intraperitoneal chemotherapy |
| #4 Systemic therapy |  | Adjuvant chemotherapy OR Neoadjuvant chemotherapy OR Neoadjuvant therapy OR Perioperative chemotherapy OR Perioperative systemic therapy OR Adjuvant systemic therapy OR neoadjuvant systemic therapy OR Systemic therapy OR Chemotherapy OR Cancer chemotherapy |
| #5 Outcomes |  | Survival OR Survival rate OR Disease-free survival OR Overall survival OR Survival analysis |
| Search: #1 Free search AND #2 Free search AND #3 Free search AND #4 Free search AND #5 Free search) | | |

| Supplementary Table 1 – Search strategy PubMed | | |
| --- | --- | --- |
|  | MeSH-term | Free search |
| #1 Colorectal carcinoma | Colorectal Neoplasms OR Colonic Neoplasms OR Rectal Neoplasms | Colorectal cancer OR Colorectal carcinoma OR Colon cancer OR Colon carcinoma OR Rectal cancer OR Rectal carcinoma OR Colorectal malignancies OR Colorectal neoplasia OR Colorectal neoplasm OR Rectal malignancies OR Rectal neoplasia OR Rectal neoplasm |
| #2 Peritoneal metastases |  | Peritoneal metastases OR Peritoneal metastasis OR Peritoneal carcinomatosis OR Peritoneum OR Carcinomatosis OR Peritoneal surface malignancies |
| #3 CRS-HIPEC | Cytoreduction Surgical Procedures OR Hyperthermic Intraperitoneal Chemotherapy | CRS OR Cytoreductive surgery OR Debulking OR Cytoreduction OR Hyperthermic intraperitoneal chemotherapy OR HIPEC OR Intraperitoneal chemotherapy |
| #4 Systemic therapy | Chemotherapy, Adjuvant OR Neoadjuvant Therapy | Perioperative chemotherapy OR Adjuvant chemotherapy OR Neoadjuvant chemotherapy OR Perioperative systemic therapy OR Adjuvant systemic therapy OR Neoadjuvant systemic therapy OR Systemic therapy OR Chemotherapy |
| #5 Outcomes | Survival OR Disease-Free Survival OR Survival Analysis | Survival OR Disease-free survival OR Survival analysis |
| Search: (#1 MeSH-term OR #1 Free search) AND (#2 MeSH-term OR #2 Free search) AND (#3 MeSH-term OR #3 Free search) AND (#4 MeSH-term OR #4 Free search) AND (#5 MeSH-term OR #5 Free search) | | |
